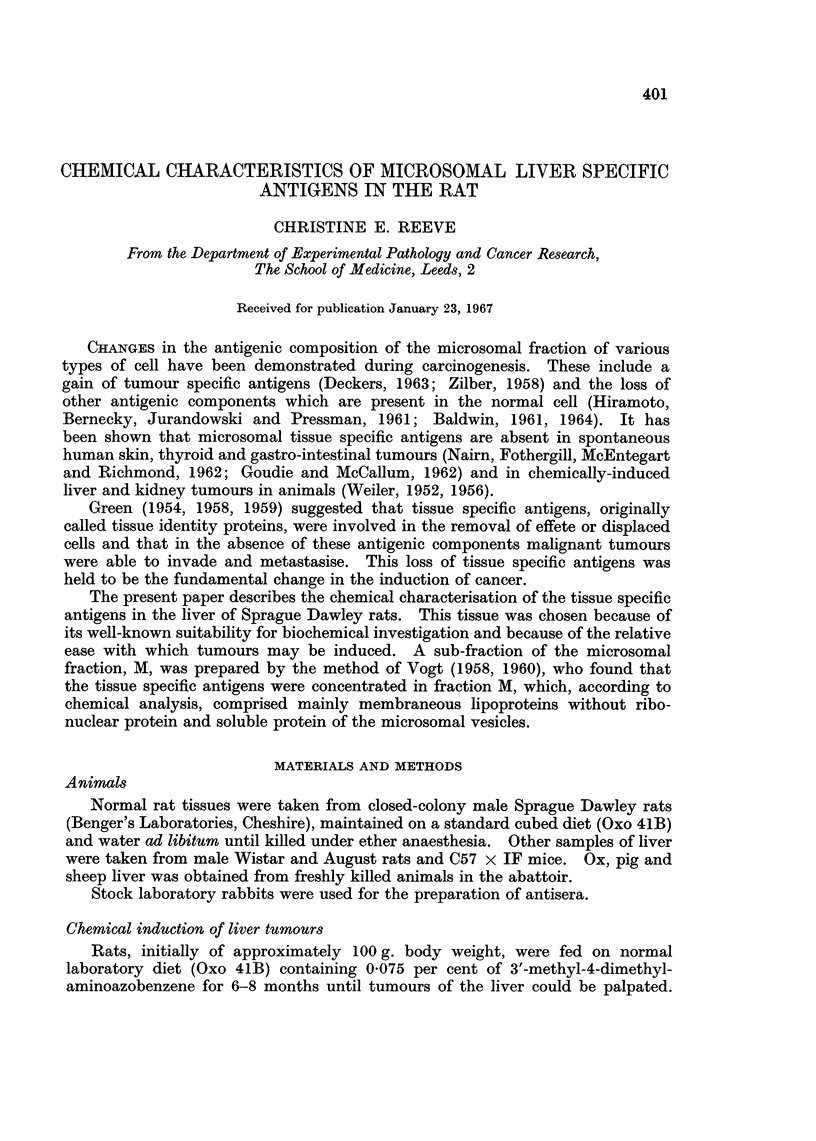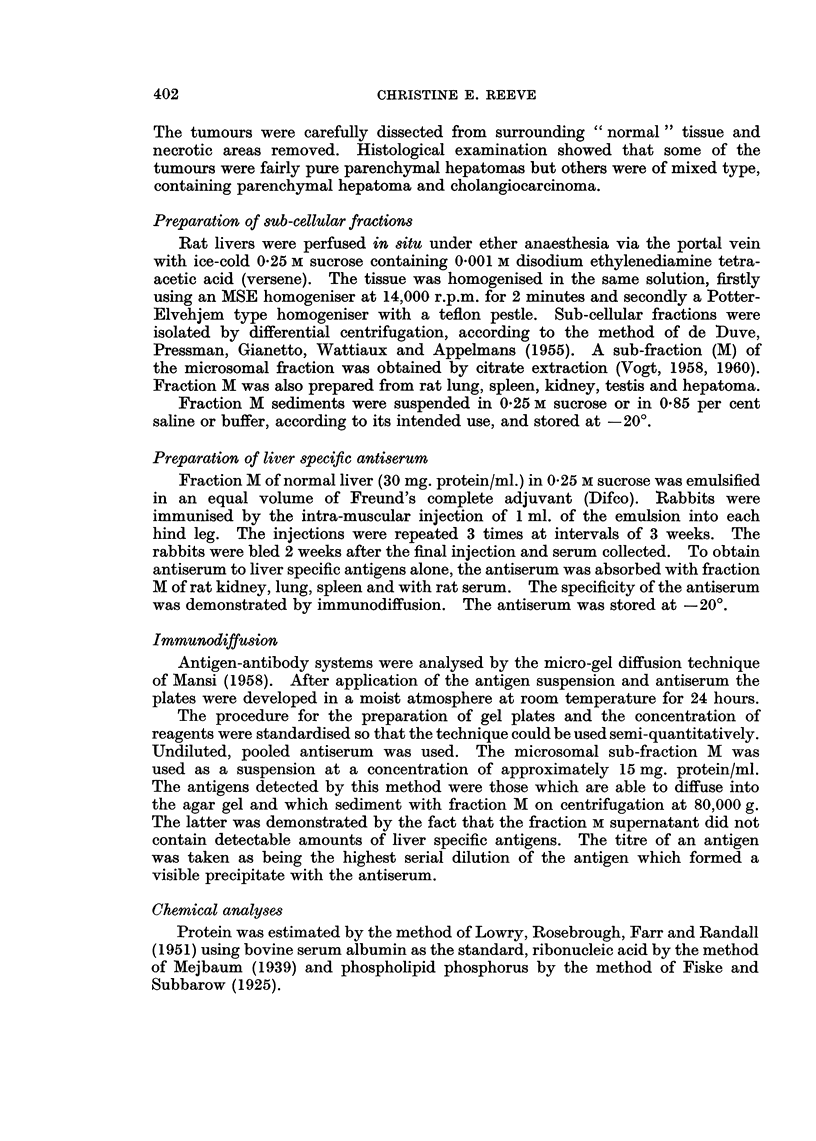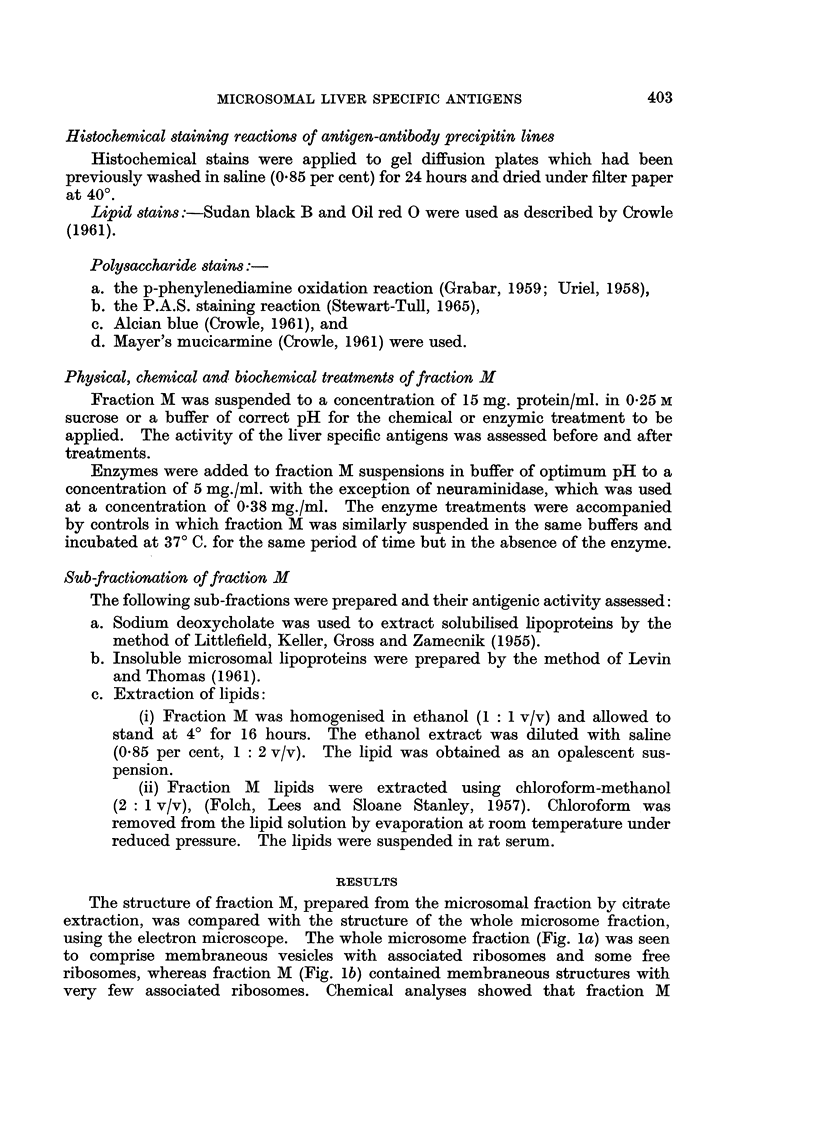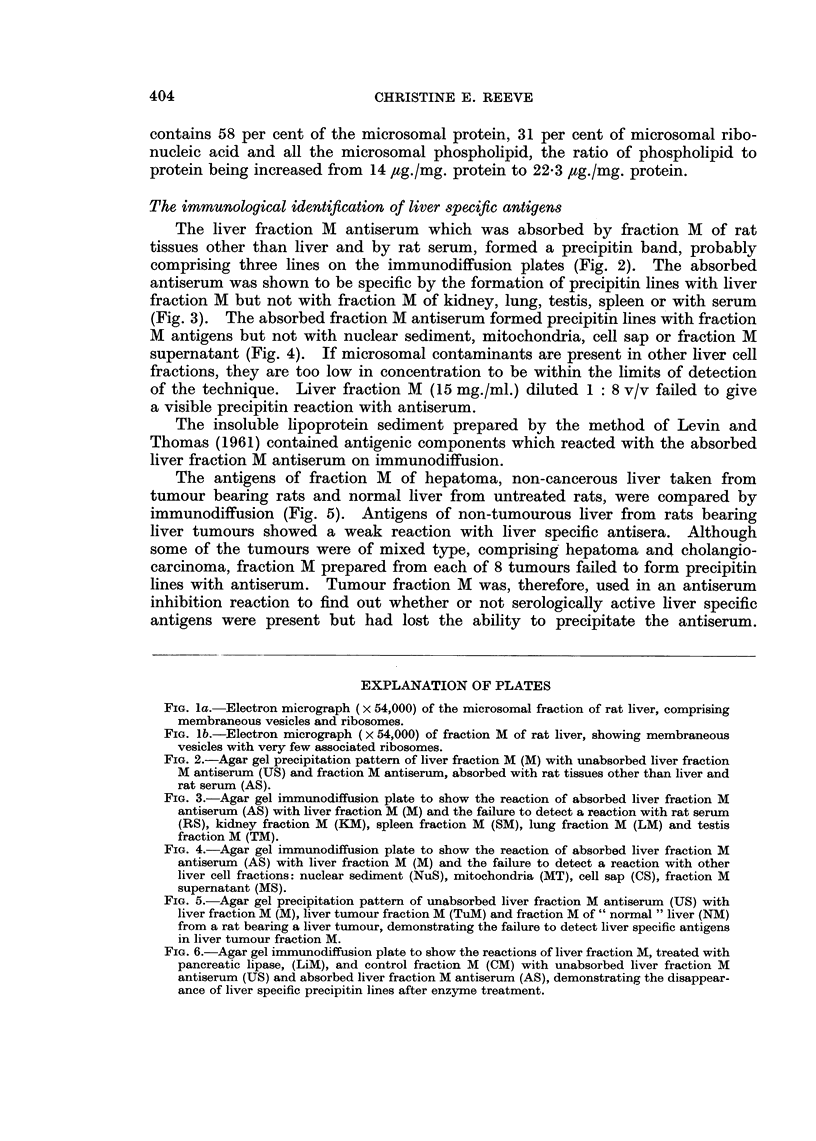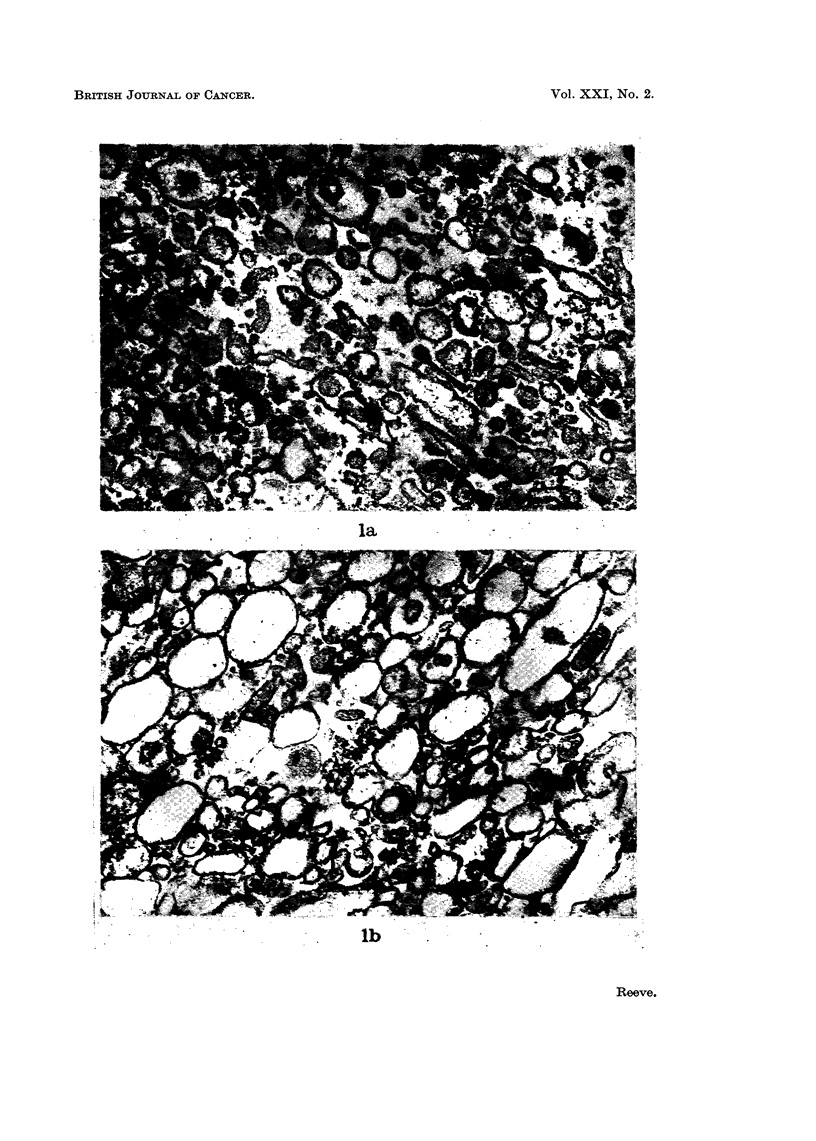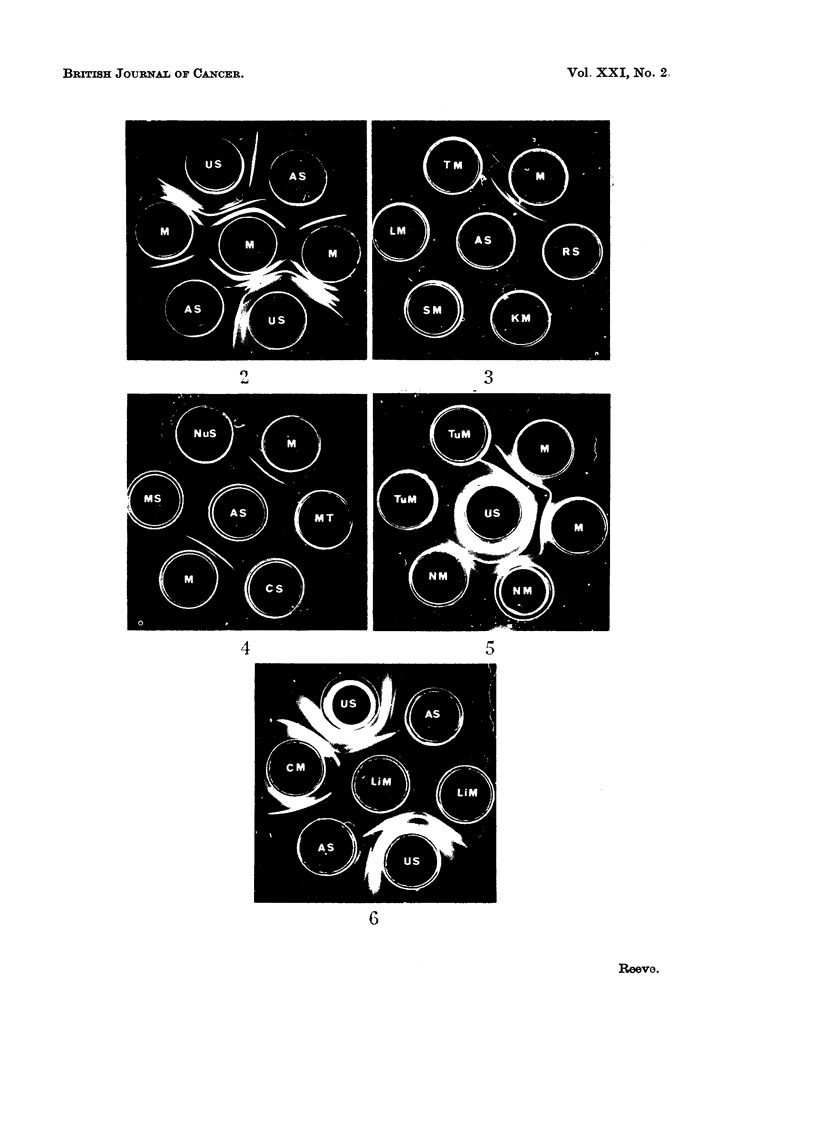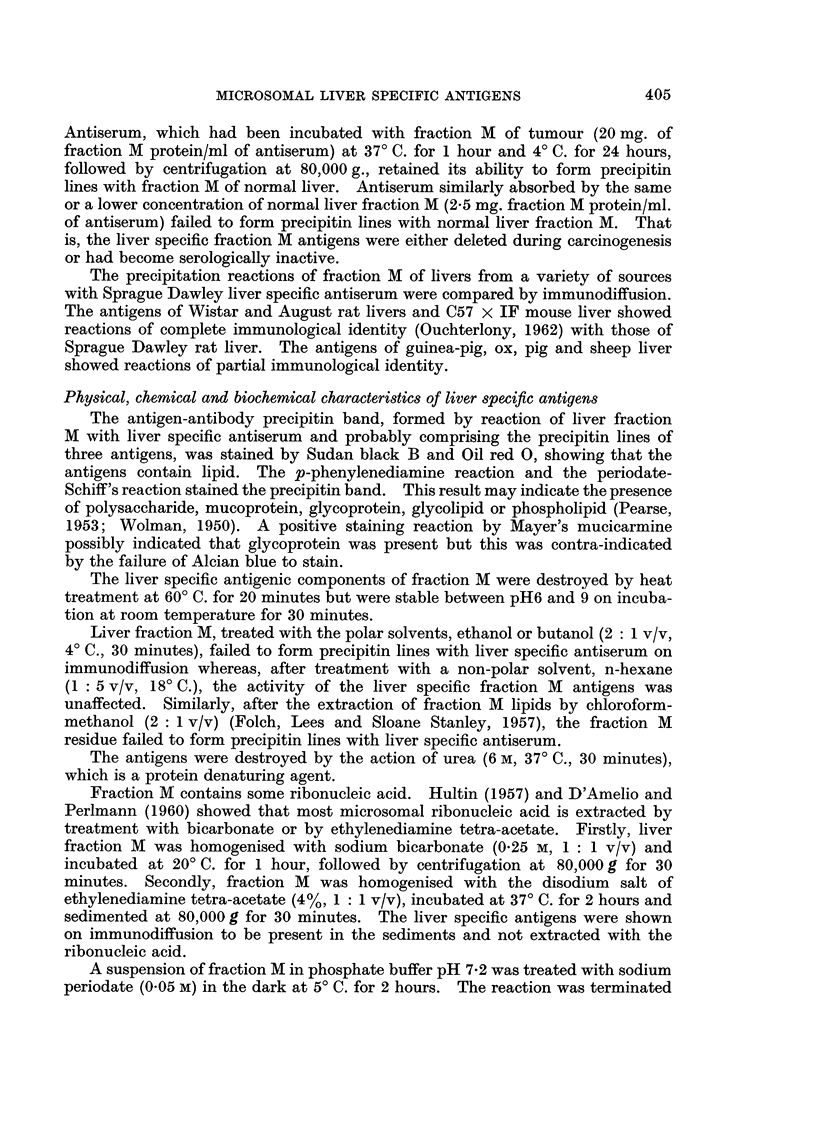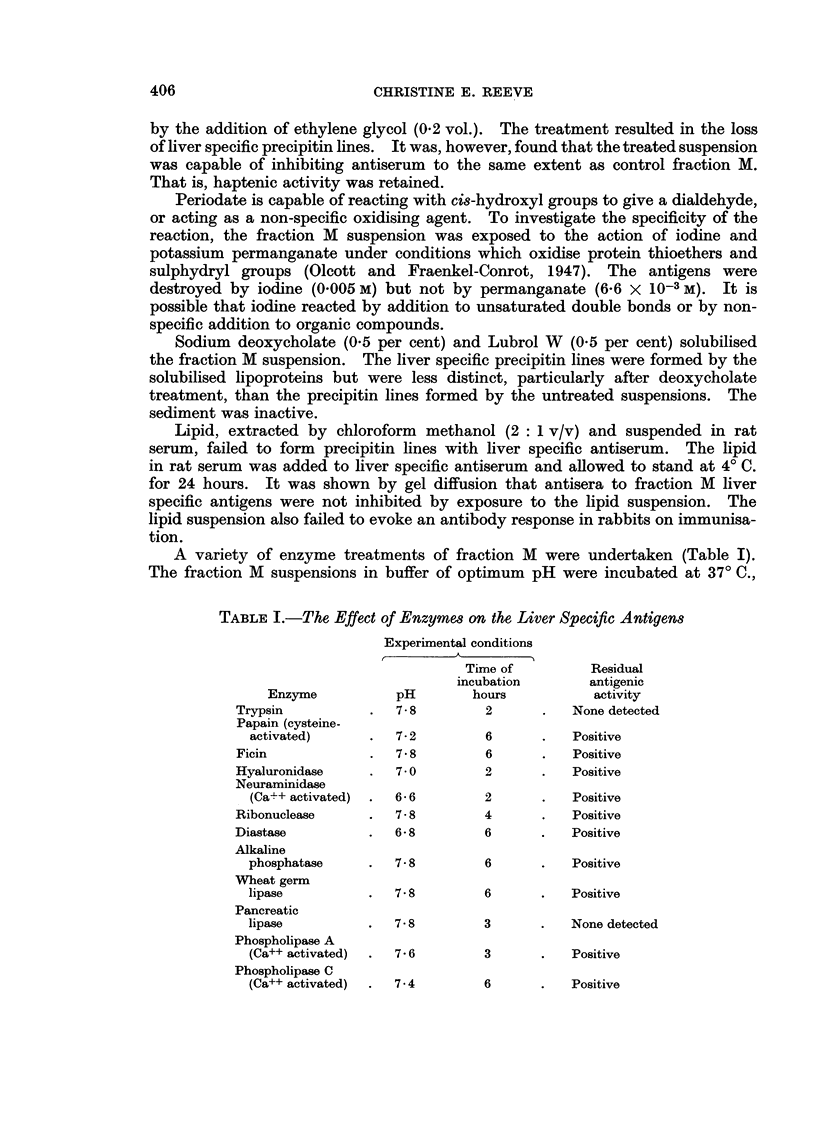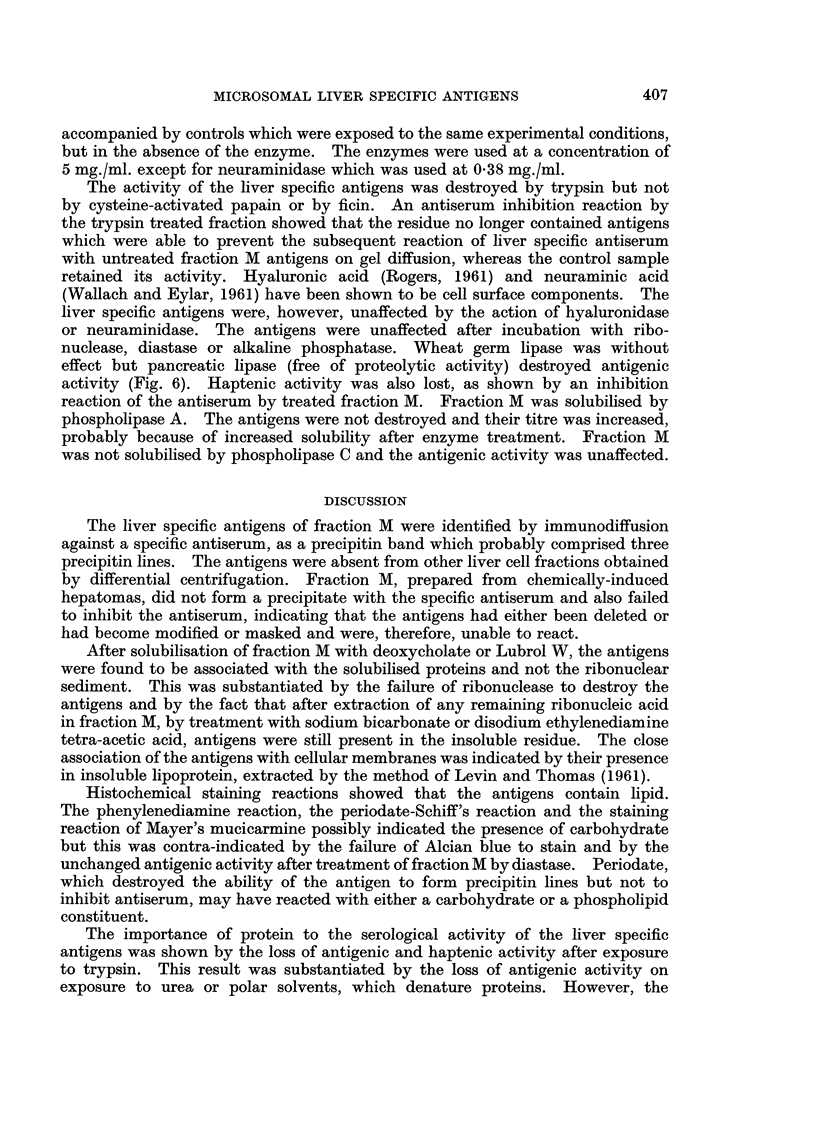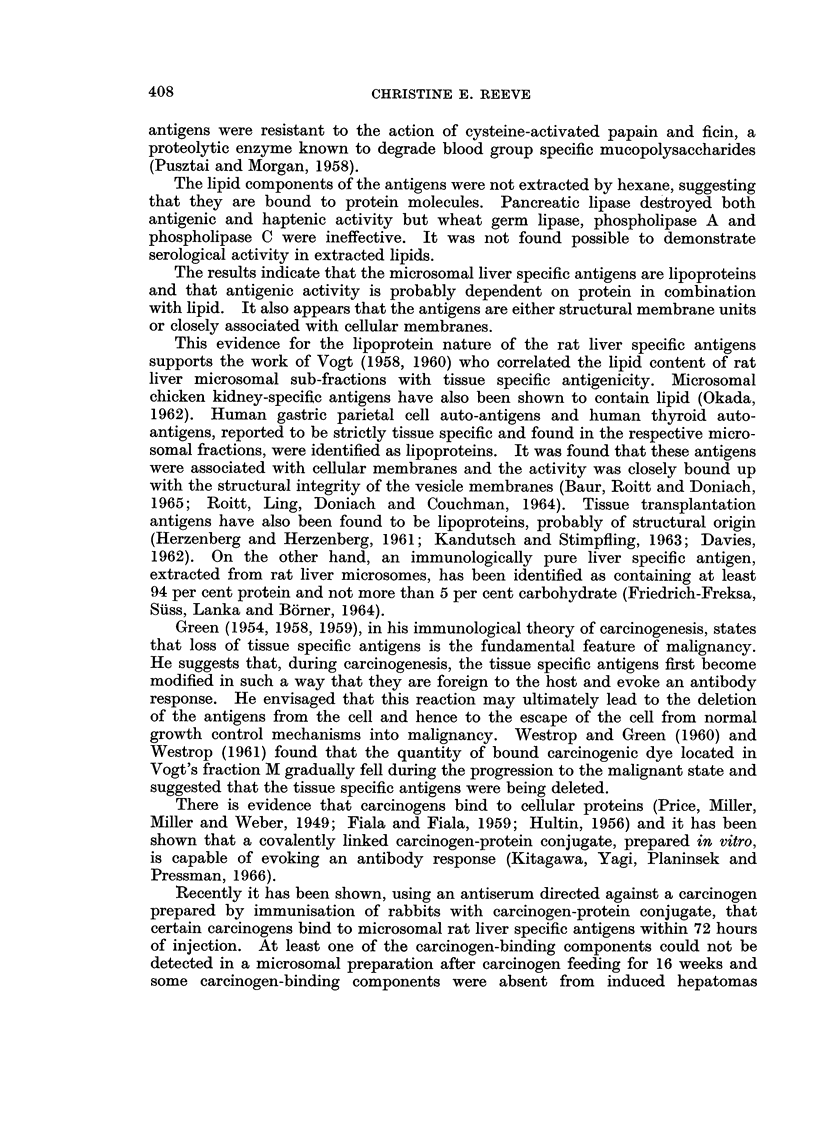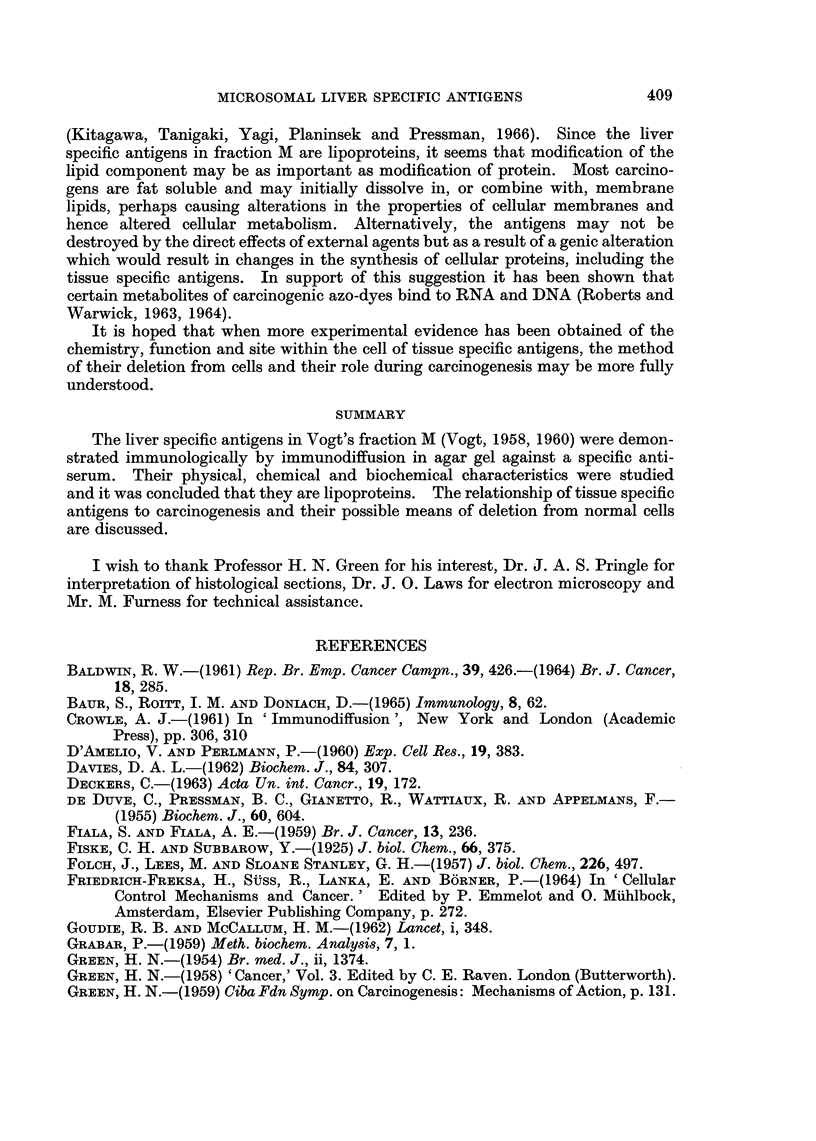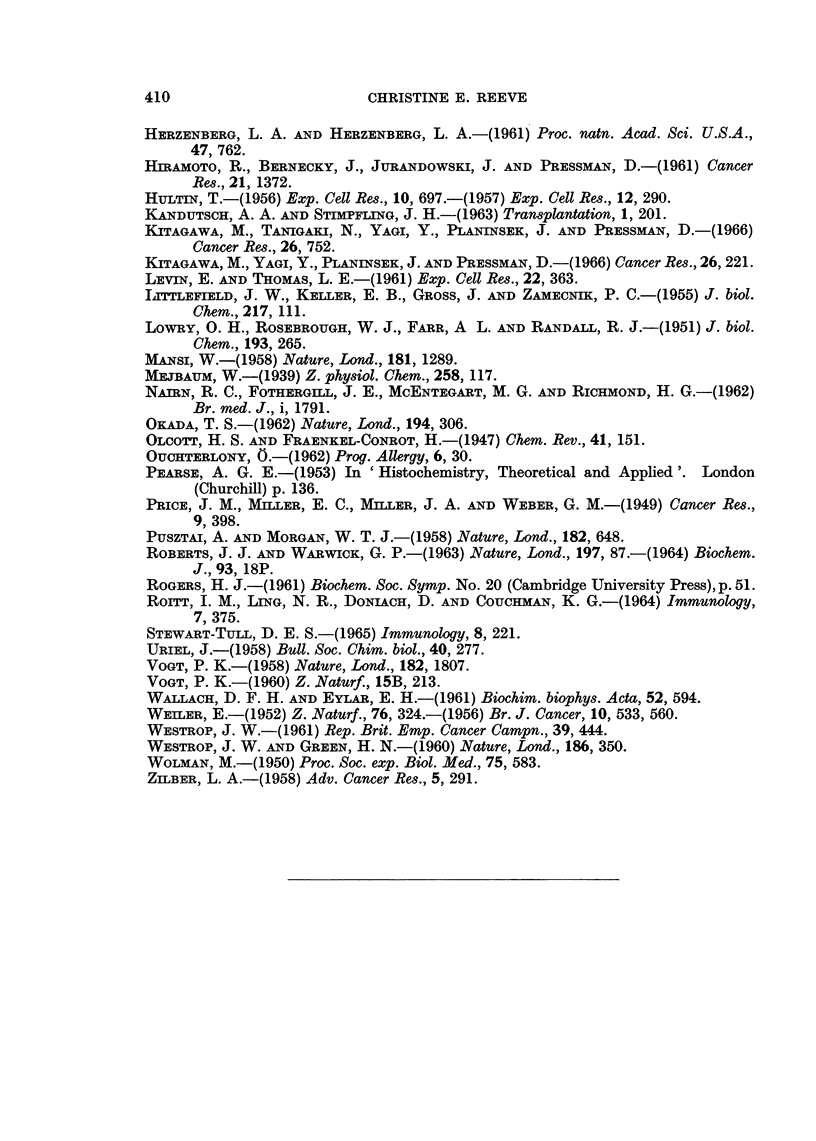# Chemical characteristics of microsomal liver specific antigens in the rat.

**DOI:** 10.1038/bjc.1967.46

**Published:** 1967-06

**Authors:** C. E. Reeve

## Abstract

**Images:**


### CHEMICAL CHARACTERISTICS OF MICROSOMAL LIVER SPECIFIC ANTIGENS IN THE RAT

CHRISTINE E. REEVE

*From the Department of Experimental Pathology and Cancer Research, The School of Medicine, Leeds, 2*

Received for publication January 23, 1967

CHANGES in the antigenic composition of the microsomal fraction of various types of cell have been demonstrated during carcinogenesis. These include a gain of tumour specific antigens (Deckers, 1963; Zilber, 1958) and the loss of other antigenic components which are present in the normal cell (Hiramoto, Bernecky, Jurandowski and Pressman, 1961; Baldwin, 1961, 1964). It has been shown that microsomal tissue specific antigens are absent in spontaneous human skin, thyroid and gastro-intestinal tumours (Nairn, Fothergill, McEntegart and Richmond, 1962; Goudie and McCallum, 1962) and in chemically-induced liver and kidney tumours in animals (Weiler, 1952, 1956).

Green (1954, 1958, 1959) suggested that tissue specific antigens, originally called tissue identity proteins, were involved in the removal of effete or displaced cells and that in the absence of these antigenic components malignant tumours were able to invade and metastasise. This loss of tissue specific antigens was held to be the fundamental change in the induction of cancer.

The present paper describes the chemical characterisation of the tissue specific antigens in the liver of Sprague Dawley rats. This tissue was chosen because of its well-known suitability for biochemical investigation and because of the relative ease with which tumours may be induced. A sub-fraction of the microsomal fraction, M, was prepared by the method of Vogt (1958, 1960), who found that the tissue specific antigens were concentrated in fraction M, which, according to chemical analysis, comprised mainly membraneous lipoproteins without ribonuclear protein and soluble protein of the microsomal vesicles.

### MATERIALS AND METHODS

### *Animals*

Normal rat tissues were taken from closed-colony male Sprague Dawley rats (Benger's Laboratories, Cheshire), maintained on a standard cubed diet (Oxo 41B) and water *ad libitum* until killed under ether anaesthesia. Other samples of liver were taken from male Wistar and August rats and C57 × IF mice. Ox, pig and sheep liver was obtained from freshly killed animals in the abattoir.

Stock laboratory rabbits were used for the preparation of antisera.

### *Chemical induction of liver tumours*

Rats, initially of approximately 100 g. body weight, were fed on normal laboratory diet (Oxo 41B) containing 0·075 per cent of 3′-methyl-4-dimethylaminoazobenzene for 6–8 months until tumours of the liver could be palpated.

The tumours were carefully dissected from surrounding " normal " tissue and necrotic areas removed. Histological examination showed that some of the tumours were fairly pure parenchymal hepatomas but others were of mixed type, containing parenchymal hepatoma and cholangiocarcinoma.

*Preparation of sub-cellular fractions*

Rat livers were perfused *in situ* under ether anaesthesia via the portal vein with ice-cold 0·25 M sucrose containing 0·001 M disodium ethylenediamine tetra-acetic acid (versene). The tissue was homogenised in the same solution, firstly using an MSE homogeniser at 14,000 r.p.m. for 2 minutes and secondly a Potter-Elvehjem type homogeniser with a teflon pestle. Sub-cellular fractions were isolated by differential centrifugation, according to the method of de Duve, Pressman, Gianetto, Wattiaux and Appelmans (1955). A sub-fraction (M) of the microsomal fraction was obtained by citrate extraction (Vogt, 1958, 1960). Fraction M was also prepared from rat lung, spleen, kidney, testis and hepatoma.

Fraction M sediments were suspended in 0·25 M sucrose or in 0·85 per cent saline or buffer, according to its intended use, and stored at $-20°$.

*Preparation of liver specific antiserum*

Fraction M of normal liver (30 mg. protein/ml.) in 0·25 M sucrose was emulsified in an equal volume of Freund's complete adjuvant (Difco). Rabbits were immunised by the intra-muscular injection of 1 ml. of the emulsion into each hind leg. The injections were repeated 3 times at intervals of 3 weeks. The rabbits were bled 2 weeks after the final injection and serum collected. To obtain antiserum to liver specific antigens alone, the antiserum was absorbed with fraction M of rat kidney, lung, spleen and with rat serum. The specificity of the antiserum was demonstrated by immunodiffusion. The antiserum was stored at $-20°$.

*Immunodiffusion*

Antigen-antibody systems were analysed by the micro-gel diffusion technique of Mansi (1958). After application of the antigen suspension and antiserum the plates were developed in a moist atmosphere at room temperature for 24 hours.

The procedure for the preparation of gel plates and the concentration of reagents were standardised so that the technique could be used semi-quantitatively. Undiluted, pooled antiserum was used. The microsomal sub-fraction M was used as a suspension at a concentration of approximately 15 mg. protein/ml. The antigens detected by this method were those which are able to diffuse into the agar gel and which sediment with fraction M on centrifugation at 80,000 g. The latter was demonstrated by the fact that the fraction M supernatant did not contain detectable amounts of liver specific antigens. The titre of an antigen was taken as being the highest serial dilution of the antigen which formed a visible precipitate with the antiserum.

*Chemical analyses*

Protein was estimated by the method of Lowry, Rosebrough, Farr and Randall (1951) using bovine serum albumin as the standard, ribonucleic acid by the method of Mejbaum (1939) and phospholipid phosphorus by the method of Fiske and Subbarow (1925).

*Histochemical staining reactions of antigen-antibody precipitin lines*

Histochemical stains were applied to gel diffusion plates which had been previously washed in saline (0·85 per cent) for 24 hours and dried under filter paper at 40°.

*Lipid stains:*—Sudan black B and Oil red O were used as described by Crowle (1961).

*Polysaccharide stains:*—

a. the p-phenylenediamine oxidation reaction (Grabar, 1959; Uriel, 1958),
b. the P.A.S. staining reaction (Stewart-Tull, 1965),
c. Alcian blue (Crowle, 1961), and
d. Mayer's mucicarmine (Crowle, 1961) were used.

*Physical, chemical and biochemical treatments of fraction M*

Fraction M was suspended to a concentration of 15 mg. protein/ml. in 0·25 M sucrose or a buffer of correct pH for the chemical or enzymic treatment to be applied. The activity of the liver specific antigens was assessed before and after treatments.

Enzymes were added to fraction M suspensions in buffer of optimum pH to a concentration of 5 mg./ml. with the exception of neuraminidase, which was used at a concentration of 0·38 mg./ml. The enzyme treatments were accompanied by controls in which fraction M was similarly suspended in the same buffers and incubated at 37° C. for the same period of time but in the absence of the enzyme.

*Sub-fractionation of fraction M*

The following sub-fractions were prepared and their antigenic activity assessed:

a. Sodium deoxycholate was used to extract solubilised lipoproteins by the method of Littlefield, Keller, Gross and Zamecnik (1955).
b. Insoluble microsomal lipoproteins were prepared by the method of Levin and Thomas (1961).
c. Extraction of lipids:

(i) Fraction M was homogenised in ethanol (1 : 1 v/v) and allowed to stand at 4° for 16 hours. The ethanol extract was diluted with saline (0·85 per cent, 1 : 2 v/v). The lipid was obtained as an opalescent suspension.

(ii) Fraction M lipids were extracted using chloroform-methanol (2 : 1 v/v), (Folch, Lees and Sloane Stanley, 1957). Chloroform was removed from the lipid solution by evaporation at room temperature under reduced pressure. The lipids were suspended in rat serum.

### RESULTS

The structure of fraction M, prepared from the microsomal fraction by citrate extraction, was compared with the structure of the whole microsome fraction, using the electron microscope. The whole microsome fraction (Fig. 1*a*) was seen to comprise membraneous vesicles with associated ribosomes and some free ribosomes, whereas fraction M (Fig. 1*b*) contained membraneous structures with very few associated ribosomes. Chemical analyses showed that fraction M

contains 58 per cent of the microsomal protein, 31 per cent of microsomal ribonucleic acid and all the microsomal phospholipid, the ratio of phospholipid to protein being increased from 14 $\mu$g./mg. protein to 22·3 $\mu$g./mg. protein.

*The immunological identification of liver specific antigens*

The liver fraction M antiserum which was absorbed by fraction M of rat tissues other than liver and by rat serum, formed a precipitin band, probably comprising three lines on the immunodiffusion plates (Fig. 2). The absorbed antiserum was shown to be specific by the formation of precipitin lines with liver fraction M but not with fraction M of kidney, lung, testis, spleen or with serum (Fig. 3). The absorbed fraction M antiserum formed precipitin lines with fraction M antigens but not with nuclear sediment, mitochondria, cell sap or fraction M supernatant (Fig. 4). If microsomal contaminants are present in other liver cell fractions, they are too low in concentration to be within the limits of detection of the technique. Liver fraction M (15 mg./ml.) diluted 1 : 8 v/v failed to give a visible precipitin reaction with antiserum.

The insoluble lipoprotein sediment prepared by the method of Levin and Thomas (1961) contained antigenic components which reacted with the absorbed liver fraction M antiserum on immunodiffusion.

The antigens of fraction M of hepatoma, non-cancerous liver taken from tumour bearing rats and normal liver from untreated rats, were compared by immunodiffusion (Fig. 5). Antigens of non-tumourous liver from rats bearing liver tumours showed a weak reaction with liver specific antisera. Although some of the tumours were of mixed type, comprising hepatoma and cholangiocarcinoma, fraction M prepared from each of 8 tumours failed to form precipitin lines with antiserum. Tumour fraction M was, therefore, used in an antiserum inhibition reaction to find out whether or not serologically active liver specific antigens were present but had lost the ability to precipitate the antiserum.

---

EXPLANATION OF PLATES

FIG. 1*a*.—Electron micrograph ($\times$ 54,000) of the microsomal fraction of rat liver, comprising membraneous vesicles and ribosomes.

FIG. 1*b*.—Electron micrograph ($\times$ 54,000) of fraction M of rat liver, showing membraneous vesicles with very few associated ribosomes.

FIG. 2.—Agar gel precipitation pattern of liver fraction M (M) with unabsorbed liver fraction M antiserum (US) and fraction M antiserum, absorbed with rat tissues other than liver and rat serum (AS).

FIG. 3.—Agar gel immunodiffusion plate to show the reaction of absorbed liver fraction M antiserum (AS) with liver fraction M (M) and the failure to detect a reaction with rat serum (RS), kidney fraction M (KM), spleen fraction M (SM), lung fraction M (LM) and testis fraction M (TM).

FIG. 4.—Agar gel immunodiffusion plate to show the reaction of absorbed liver fraction M antiserum (AS) with liver fraction M (M) and the failure to detect a reaction with other liver cell fractions: nuclear sediment (NuS), mitochondria (MT), cell sap (CS), fraction M supernatant (MS).

FIG. 5.—Agar gel precipitation pattern of unabsorbed liver fraction M antiserum (US) with liver fraction M (M), liver tumour fraction M (TuM) and fraction M of " normal " liver (NM) from a rat bearing a liver tumour, demonstrating the failure to detect liver specific antigens in liver tumour fraction M.

FIG. 6.—Agar gel immunodiffusion plate to show the reactions of liver fraction M, treated with pancreatic lipase, (LiM), and control fraction M (CM) with unabsorbed liver fraction M antiserum (US) and absorbed liver fraction M antiserum (AS), demonstrating the disappearance of liver specific precipitin lines after enzyme treatment.

1a

1b

Reeve.

US
AS
M
M
M
AS
US

2

TM
M
LM
AS
RS
SM
KM

3

NuS
M
MS
AS
MT
M
CS

4

TuM
M
TuM
US
M
NM
NM

5

US
AS
CM
LiM
LiM
AS
US

6

Reeve.

Antiserum, which had been incubated with fraction M of tumour (20 mg. of fraction M protein/ml of antiserum) at 37° C. for 1 hour and 4° C. for 24 hours, followed by centrifugation at 80,000 g., retained its ability to form precipitin lines with fraction M of normal liver. Antiserum similarly absorbed by the same or a lower concentration of normal liver fraction M (2·5 mg. fraction M protein/ml. of antiserum) failed to form precipitin lines with normal liver fraction M. That is, the liver specific fraction M antigens were either deleted during carcinogenesis or had become serologically inactive.

The precipitation reactions of fraction M of livers from a variety of sources with Sprague Dawley liver specific antiserum were compared by immunodiffusion. The antigens of Wistar and August rat livers and C57 × IF mouse liver showed reactions of complete immunological identity (Ouchterlony, 1962) with those of Sprague Dawley rat liver. The antigens of guinea-pig, ox, pig and sheep liver showed reactions of partial immunological identity.

*Physical, chemical and biochemical characteristics of liver specific antigens*

The antigen-antibody precipitin band, formed by reaction of liver fraction M with liver specific antiserum and probably comprising the precipitin lines of three antigens, was stained by Sudan black B and Oil red O, showing that the antigens contain lipid. The *p*-phenylenediamine reaction and the periodate-Schiff's reaction stained the precipitin band. This result may indicate the presence of polysaccharide, mucoprotein, glycoprotein, glycolipid or phospholipid (Pearse, 1953; Wolman, 1950). A positive staining reaction by Mayer's mucicarmine possibly indicated that glycoprotein was present but this was contra-indicated by the failure of Alcian blue to stain.

The liver specific antigenic components of fraction M were destroyed by heat treatment at 60° C. for 20 minutes but were stable between pH6 and 9 on incubation at room temperature for 30 minutes.

Liver fraction M, treated with the polar solvents, ethanol or butanol (2 : 1 v/v, 4° C., 30 minutes), failed to form precipitin lines with liver specific antiserum on immunodiffusion whereas, after treatment with a non-polar solvent, n-hexane (1 : 5 v/v, 18° C.), the activity of the liver specific fraction M antigens was unaffected. Similarly, after the extraction of fraction M lipids by chloroform-methanol (2 : 1 v/v) (Folch, Lees and Sloane Stanley, 1957), the fraction M residue failed to form precipitin lines with liver specific antiserum.

The antigens were destroyed by the action of urea (6 M, 37° C., 30 minutes), which is a protein denaturing agent.

Fraction M contains some ribonucleic acid. Hultin (1957) and D'Amelio and Perlmann (1960) showed that most microsomal ribonucleic acid is extracted by treatment with bicarbonate or by ethylenediamine tetra-acetate. Firstly, liver fraction M was homogenised with sodium bicarbonate (0·25 M, 1 : 1 v/v) and incubated at 20° C. for 1 hour, followed by centrifugation at 80,000 *g* for 30 minutes. Secondly, fraction M was homogenised with the disodium salt of ethylenediamine tetra-acetate (4%, 1 : 1 v/v), incubated at 37° C. for 2 hours and sedimented at 80,000 *g* for 30 minutes. The liver specific antigens were shown on immunodiffusion to be present in the sediments and not extracted with the ribonucleic acid.

A suspension of fraction M in phosphate buffer pH 7·2 was treated with sodium periodate (0·05 M) in the dark at 5° C. for 2 hours. The reaction was terminated by the addition of ethylene glycol (0·2 vol.). The treatment resulted in the loss of liver specific precipitin lines. It was, however, found that the treated suspension was capable of inhibiting antiserum to the same extent as control fraction M. That is, haptenic activity was retained.

Periodate is capable of reacting with *cis*-hydroxyl groups to give a dialdehyde, or acting as a non-specific oxidising agent. To investigate the specificity of the reaction, the fraction M suspension was exposed to the action of iodine and potassium permanganate under conditions which oxidise protein thioethers and sulphydryl groups (Olcott and Fraenkel-Conrot, 1947). The antigens were destroyed by iodine (0·005 M) but not by permanganate ($6 \cdot 6 \times 10^{-3}$ M). It is possible that iodine reacted by addition to unsaturated double bonds or by non-specific addition to organic compounds.

Sodium deoxycholate (0·5 per cent) and Lubrol W (0·5 per cent) solubilised the fraction M suspension. The liver specific precipitin lines were formed by the solubilised lipoproteins but were less distinct, particularly after deoxycholate treatment, than the precipitin lines formed by the untreated suspensions. The sediment was inactive.

Lipid, extracted by chloroform methanol (2 : 1 v/v) and suspended in rat serum, failed to form precipitin lines with liver specific antiserum. The lipid in rat serum was added to liver specific antiserum and allowed to stand at 4° C. for 24 hours. It was shown by gel diffusion that antisera to fraction M liver specific antigens were not inhibited by exposure to the lipid suspension. The lipid suspension also failed to evoke an antibody response in rabbits on immunisation.

A variety of enzyme treatments of fraction M were undertaken (Table I). The fraction M suspensions in buffer of optimum pH were incubated at 37° C.,

TABLE I.—*The Effect of Enzymes on the Liver Specific Antigens*

| Enzyme | Experimental conditions: pH | Experimental conditions: Time of incubation hours | Residual antigenic activity |
|---|---|---|---|
| Trypsin | 7·8 | 2 | None detected |
| Papain (cysteine-activated) | 7·2 | 6 | Positive |
| Ficin | 7·8 | 6 | Positive |
| Hyaluronidase | 7·0 | 2 | Positive |
| Neuraminidase ($Ca^{++}$ activated) | 6·6 | 2 | Positive |
| Ribonuclease | 7·8 | 4 | Positive |
| Diastase | 6·8 | 6 | Positive |
| Alkaline phosphatase | 7·8 | 6 | Positive |
| Wheat germ lipase | 7·8 | 6 | Positive |
| Pancreatic lipase | 7·8 | 3 | None detected |
| Phospholipase A ($Ca^{++}$ activated) | 7·6 | 3 | Positive |
| Phospholipase C ($Ca^{++}$ activated) | 7·4 | 6 | Positive |

accompanied by controls which were exposed to the same experimental conditions, but in the absence of the enzyme. The enzymes were used at a concentration of 5 mg./ml. except for neuraminidase which was used at 0·38 mg./ml.

The activity of the liver specific antigens was destroyed by trypsin but not by cysteine-activated papain or by ficin. An antiserum inhibition reaction by the trypsin treated fraction showed that the residue no longer contained antigens which were able to prevent the subsequent reaction of liver specific antiserum with untreated fraction M antigens on gel diffusion, whereas the control sample retained its activity. Hyaluronic acid (Rogers, 1961) and neuraminic acid (Wallach and Eylar, 1961) have been shown to be cell surface components. The liver specific antigens were, however, unaffected by the action of hyaluronidase or neuraminidase. The antigens were unaffected after incubation with ribonuclease, diastase or alkaline phosphatase. Wheat germ lipase was without effect but pancreatic lipase (free of proteolytic activity) destroyed antigenic activity (Fig. 6). Haptenic activity was also lost, as shown by an inhibition reaction of the antiserum by treated fraction M. Fraction M was solubilised by phospholipase A. The antigens were not destroyed and their titre was increased, probably because of increased solubility after enzyme treatment. Fraction M was not solubilised by phospholipase C and the antigenic activity was unaffected.

### DISCUSSION

The liver specific antigens of fraction M were identified by immunodiffusion against a specific antiserum, as a precipitin band which probably comprised three precipitin lines. The antigens were absent from other liver cell fractions obtained by differential centrifugation. Fraction M, prepared from chemically-induced hepatomas, did not form a precipitate with the specific antiserum and also failed to inhibit the antiserum, indicating that the antigens had either been deleted or had become modified or masked and were, therefore, unable to react.

After solubilisation of fraction M with deoxycholate or Lubrol W, the antigens were found to be associated with the solubilised proteins and not the ribonuclear sediment. This was substantiated by the failure of ribonuclease to destroy the antigens and by the fact that after extraction of any remaining ribonucleic acid in fraction M, by treatment with sodium bicarbonate or disodium ethylenediamine tetra-acetic acid, antigens were still present in the insoluble residue. The close association of the antigens with cellular membranes was indicated by their presence in insoluble lipoprotein, extracted by the method of Levin and Thomas (1961).

Histochemical staining reactions showed that the antigens contain lipid. The phenylenediamine reaction, the periodate-Schiff's reaction and the staining reaction of Mayer's mucicarmine possibly indicated the presence of carbohydrate but this was contra-indicated by the failure of Alcian blue to stain and by the unchanged antigenic activity after treatment of fraction M by diastase. Periodate, which destroyed the ability of the antigen to form precipitin lines but not to inhibit antiserum, may have reacted with either a carbohydrate or a phospholipid constituent.

The importance of protein to the serological activity of the liver specific antigens was shown by the loss of antigenic and haptenic activity after exposure to trypsin. This result was substantiated by the loss of antigenic activity on exposure to urea or polar solvents, which denature proteins. However, the antigens were resistant to the action of cysteine-activated papain and ficin, a proteolytic enzyme known to degrade blood group specific mucopolysaccharides (Pusztai and Morgan, 1958).

The lipid components of the antigens were not extracted by hexane, suggesting that they are bound to protein molecules. Pancreatic lipase destroyed both antigenic and haptenic activity but wheat germ lipase, phospholipase A and phospholipase C were ineffective. It was not found possible to demonstrate serological activity in extracted lipids.

The results indicate that the microsomal liver specific antigens are lipoproteins and that antigenic activity is probably dependent on protein in combination with lipid. It also appears that the antigens are either structural membrane units or closely associated with cellular membranes.

This evidence for the lipoprotein nature of the rat liver specific antigens supports the work of Vogt (1958, 1960) who correlated the lipid content of rat liver microsomal sub-fractions with tissue specific antigenicity. Microsomal chicken kidney-specific antigens have also been shown to contain lipid (Okada, 1962). Human gastric parietal cell auto-antigens and human thyroid auto-antigens, reported to be strictly tissue specific and found in the respective microsomal fractions, were identified as lipoproteins. It was found that these antigens were associated with cellular membranes and the activity was closely bound up with the structural integrity of the vesicle membranes (Baur, Roitt and Doniach, 1965; Roitt, Ling, Doniach and Couchman, 1964). Tissue transplantation antigens have also been found to be lipoproteins, probably of structural origin (Herzenberg and Herzenberg, 1961; Kandutsch and Stimpfling, 1963; Davies, 1962). On the other hand, an immunologically pure liver specific antigen, extracted from rat liver microsomes, has been identified as containing at least 94 per cent protein and not more than 5 per cent carbohydrate (Friedrich-Freksa, Süss, Lanka and Börner, 1964).

Green (1954, 1958, 1959), in his immunological theory of carcinogenesis, states that loss of tissue specific antigens is the fundamental feature of malignancy. He suggests that, during carcinogenesis, the tissue specific antigens first become modified in such a way that they are foreign to the host and evoke an antibody response. He envisaged that this reaction may ultimately lead to the deletion of the antigens from the cell and hence to the escape of the cell from normal growth control mechanisms into malignancy. Westrop and Green (1960) and Westrop (1961) found that the quantity of bound carcinogenic dye located in Vogt's fraction M gradually fell during the progression to the malignant state and suggested that the tissue specific antigens were being deleted.

There is evidence that carcinogens bind to cellular proteins (Price, Miller, Miller and Weber, 1949; Fiala and Fiala, 1959; Hultin, 1956) and it has been shown that a covalently linked carcinogen-protein conjugate, prepared *in vitro*, is capable of evoking an antibody response (Kitagawa, Yagi, Planinsek and Pressman, 1966).

Recently it has been shown, using an antiserum directed against a carcinogen prepared by immunisation of rabbits with carcinogen-protein conjugate, that certain carcinogens bind to microsomal rat liver specific antigens within 72 hours of injection. At least one of the carcinogen-binding components could not be detected in a microsomal preparation after carcinogen feeding for 16 weeks and some carcinogen-binding components were absent from induced hepatomas

(Kitagawa, Tanigaki, Yagi, Planinsek and Pressman, 1966). Since the liver specific antigens in fraction M are lipoproteins, it seems that modification of the lipid component may be as important as modification of protein. Most carcinogens are fat soluble and may initially dissolve in, or combine with, membrane lipids, perhaps causing alterations in the properties of cellular membranes and hence altered cellular metabolism. Alternatively, the antigens may not be destroyed by the direct effects of external agents but as a result of a genic alteration which would result in changes in the synthesis of cellular proteins, including the tissue specific antigens. In support of this suggestion it has been shown that certain metabolites of carcinogenic azo-dyes bind to RNA and DNA (Roberts and Warwick, 1963, 1964).

It is hoped that when more experimental evidence has been obtained of the chemistry, function and site within the cell of tissue specific antigens, the method of their deletion from cells and their role during carcinogenesis may be more fully understood.

### SUMMARY

The liver specific antigens in Vogt's fraction M (Vogt, 1958, 1960) were demonstrated immunologically by immunodiffusion in agar gel against a specific antiserum. Their physical, chemical and biochemical characteristics were studied and it was concluded that they are lipoproteins. The relationship of tissue specific antigens to carcinogenesis and their possible means of deletion from normal cells are discussed.

I wish to thank Professor H. N. Green for his interest, Dr. J. A. S. Pringle for interpretation of histological sections, Dr. J. O. Laws for electron microscopy and Mr. M. Furness for technical assistance.

### REFERENCES

Baldwin, R. W.—(1961) *Rep. Br. Emp. Cancer Campn.*, **39**, 426.—(1964) *Br. J. Cancer*, **18**, 285.
Baur, S., Roitt, I. M. and Doniach, D.—(1965) *Immunology*, **8**, 62.
Crowle, A. J.—(1961) In ' Immunodiffusion ', New York and London (Academic Press), pp. 306, 310
D'Amelio, V. and Perlmann, P.—(1960) *Exp. Cell Res.*, **19**, 383.
Davies, D. A. L.—(1962) *Biochem. J.*, **84**, 307.
Deckers, C.—(1963) *Acta Un. int. Cancr.*, **19**, 172.
de Duve, C., Pressman, B. C., Gianetto, R., Wattiaux, R. and Appelmans, F.—(1955) *Biochem. J.*, **60**, 604.
Fiala, S. and Fiala, A. E.—(1959) *Br. J. Cancer*, **13**, 236.
Fiske, C. H. and Subbarow, Y.—(1925) *J. biol. Chem.*, **66**, 375.
Folch, J., Lees, M. and Sloane Stanley, G. H.—(1957) *J. biol. Chem.*, **226**, 497.
Friedrich-Freksa, H., Süss, R., Lanka, E. and Börner, P.—(1964) In ' Cellular Control Mechanisms and Cancer. ' Edited by P. Emmelot and O. Mühlbock, Amsterdam, Elsevier Publishing Company, p. 272.
Goudie, R. B. and McCallum, H. M.—(1962) *Lancet*, i, 348.
Grabar, P.—(1959) *Meth. biochem. Analysis*, **7**, 1.
Green, H. N.—(1954) *Br. med. J.*, ii, 1374.
Green, H. N.—(1958) 'Cancer,' Vol. 3. Edited by C. E. Raven. London (Butterworth).
Green, H. N.—(1959) *Ciba Fdn Symp.* on Carcinogenesis: Mechanisms of Action, p. 131.

Herzenberg, L. A. and Herzenberg, L. A.—(1961) *Proc. natn. Acad. Sci. U.S.A.*, **47**, 762.
Hiramoto, R., Bernecky, J., Jurandowski, J. and Pressman, D.—(1961) *Cancer Res.*, **21**, 1372.
Hultin, T.—(1956) *Exp. Cell Res.*, **10**, 697.—(1957) *Exp. Cell Res.*, **12**, 290.
Kandutsch, A. A. and Stimpfling, J. H.—(1963) *Transplantation*, **1**, 201.
Kitagawa, M., Tanigaki, N., Yagi, Y., Planinsek, J. and Pressman, D.—(1966) *Cancer Res.*, **26**, 752.
Kitagawa, M., Yagi, Y., Planinsek, J. and Pressman, D.—(1966) *Cancer Res.*, **26**, 221.
Levin, E. and Thomas, L. E.—(1961) *Exp. Cell Res.*, **22**, 363.
Littlefield, J. W., Keller, E. B., Gross, J. and Zamecnik, P. C.—(1955) *J. biol. Chem.*, **217**, 111.
Lowry, O. H., Rosebrough, W. J., Farr, A L. and Randall, R. J.—(1951) *J. biol. Chem.*, **193**, 265.
Mansi, W.—(1958) *Nature, Lond.*, **181**, 1289.
Mejbaum, W.—(1939) *Z. physiol. Chem.*, **258**, 117.
Nairn, R. C., Fothergill, J. E., McEntegart, M. G. and Richmond, H. G.—(1962) *Br. med. J.*, i, 1791.
Okada, T. S.—(1962) *Nature, Lond.*, **194**, 306.
Olcott, H. S. and Fraenkel-Conrot, H.—(1947) *Chem. Rev.*, **41**, 151.
Ouchterlony, Ö.—(1962) *Prog. Allergy*, **6**, 30.
Pearse, A. G. E.—(1953) In ' Histochemistry, Theoretical and Applied '. London (Churchill) p. 136.
Price, J. M., Miller, E. C., Miller, J. A. and Weber, G. M.—(1949) *Cancer Res.*, **9**, 398.
Pusztai, A. and Morgan, W. T. J.—(1958) *Nature, Lond.*, **182**, 648.
Roberts, J. J. and Warwick, G. P.—(1963) *Nature, Lond.*, **197**, 87.—(1964) *Biochem. J.*, **93**, 18P.
Rogers, H. J.—(1961) *Biochem. Soc. Symp.* No. 20 (Cambridge University Press), p. 51.
Roitt, I. M., Ling, N. R., Doniach, D. and Couchman, K. G.—(1964) *Immunology*, **7**, 375.
Stewart-Tull, D. E. S.—(1965) *Immunology*, **8**, 221.
Uriel, J.—(1958) *Bull. Soc. Chim. biol.*, **40**, 277.
Vogt, P. K.—(1958) *Nature, Lond.*, **182**, 1807.
Vogt, P. K.—(1960) *Z. Naturf.*, **15B**, 213.
Wallach, D. F. H. and Eylar, E. H.—(1961) *Biochim. biophys. Acta*, **52**, 594.
Weiler, E.—(1952) *Z. Naturf.*, **76**, 324.—(1956) *Br. J. Cancer*, **10**, 533, 560.
Westrop, J. W.—(1961) *Rep. Brit. Emp. Cancer Campn.*, **39**, 444.
Westrop, J. W. and Green, H. N.—(1960) *Nature, Lond.*, **186**, 350.
Wolman, M.—(1950) *Proc. Soc. exp. Biol. Med.*, **75**, 583.
Zilber, L. A.—(1958) *Adv. Cancer Res.*, **5**, 291.